# Development and implementation of evidence-based, nurse-leading early warning model and healthcare quality improvement project for transplant-associated thrombotic microangiopathy: a mixed-methods, before-and-after study

**DOI:** 10.1186/s12912-024-02093-7

**Published:** 2024-08-07

**Authors:** Xiaoyu Zhou, Yishan Ye, Aiyun Jin, Zhengwen Pan, Zhe Xu, Shuyi Ding, Jiali Yan, Yin Cheng, Yixuan Huang, Kai Cao, Wei Xie, Jianli Zhang, Liwei Xu, Weiwei Zhou, Lihua Huang

**Affiliations:** 1 Department of Nursing, The First Affiliated Hospital, Zhejiang University School of Medical, Hangzhou, China; 2 Bone Marrow Transplantation Center, The First Affiliated Hospital, Zhejiang University School of Medical, Hangzhou, China

**Keywords:** Transplant-associated thrombotic microangiopathy, Waning model, Healthcare quality improvement project, Nurse-leading, Evidence-based

## Abstract

**Objective:**

The early identification and diagnosis of transplant-associated thrombotic microangiopathy (TA-TMA) are essential yet difficult in patients underwent hematopoietic stem cell transplantation (HSCT). To develop an evidence-based, nurse-leading early warning model for TA-TMA, and implement the healthcare quality review and improvement project.

**Methods:**

This study was a mixed-methods, before-and-after study. The early warning model was developed based on quality evidence from literature search. The healthcare quality review and improvement project mainly included baseline investigation of nurse, improvement action and effectiveness evaluation. The awareness and knowledge of early parameter of TA-TMA among nurses and the prognosis of patients underwent HSCT were compared before and after the improvement.

**Results:**

A total of 1 guideline, 1 evidence synthesis, 4 expert consensuses, 10 literature reviews, 2 diagnostic studies, and 9 case series were included in the best evidence. The early warning model including warning period, high-risk characteristics and early manifestation of TA-TMA was developed. The improvement action, including staff training and assessment, suspected TA-TMA identification and patient education, was implemented. The awareness and knowledge rate of early parameter of TA-TMA among nurses significantly improved after improvement action (100% vs. 26.7%, *P* < 0.001). The incidence of TA-TMA was similar among patients underwent HSCT before and after improvement action (2.8% vs. 1.2%, *P* = 0.643), while no fall event occurred after improvement action (0 vs. 1.2%, *P* < 0.001).

**Conclusion:**

The evidence-based early warning model and healthcare quality improvement project could enhance the awareness and knowledge of TA-TMA among healthcare providers and might improve the prognosis of patients diagnosed with TA-TMA.

## Introduction

Hematopoietic stem cell transplant-associated thrombotic microangiopathy (TA-TMA) is a syndrome of multifactorial endothelial injury that damages the kidney and other organs after transplantation [1]. It was first reported by Powles et al. in 1980 [2] with the onset of renal failure and central nervous system dysfunction such as seizure, stroke or encephalopathy, accompanied by hypertension, hemolytic anemia and consumptive thrombocytopenia in the absence of coagulopathy. The classic hallmarks of the syndrome including platelet consumption, hemolysis, and organ dysfunction, particularly the kidney suggested the manifestation diversity of TA-TMA. Furthermore, mainly due to the overlaps of symptom with other conditions and unrevealed pathological mechanism, well-established diagnostic criteria were lacking, resulting in the greatly varied incidence of TA-TMA after hematopoietic stem cell transplantation (HSCT) between 0.5% and 75% [1, 3, 4]. TA-TMA could be life-threating, mostly attribute to the multi-system involvement. The mortality rate of patients was up to 60%-100% without timely and effective treatment [5, 6]. Therefore, it is of great importance to implement optimal diagnosis and treatment strategy to improve the prognosis of patients underwent HSCT.

The three-hit hypothesis proposed by Young J.A. etc [7]. was widely accepted that the endothelial injury leads to an increase in pro-inflammatory cytokines and procoagulant factors that further promote tissue damage. The disruption of the damage might be the key in the prevention and treatment of TA-TMA. Previous studies have reported the discontinuation of cyclosporine was associated with better prognosis of patients without vascular endothelial injury or in the early stage of injury [8, 9]. Hence, the early identification and diagnosis of TA-TMA and subsequent treatment and monitor are essential, yet difficult in patients underwent HSCT.

Though studies on TA-TMA have enabled healthcare providers to be vigilant, the identified risk factor, such as gender, primary disease, conditioning intensity, infection and graft-versus-host disease (GVHD) prophylaxis, varied in different studies [10, 11]. The available prediction model mostly was constructed from single-center data including a relatively small population [12, 13], which might limit its application in the clinical practice. Hence, this study aims to develop an evidence-based model consisting of parameters for TA-TMA, and implement healthcare quality review and improvement, so as to improve the quality of medical care and the prognosis of patients (e.g. the incidence of falls) underwent HSCT.

## Methods

### Study design

This study was a mixed-methods, before-and-after study, with two work streams. The first work stream was to develop an early warning model based on quality evidence from literature search and review. The second work stream was to implement healthcare quality review and improvement, mainly including baseline investigation, improvement action and effectiveness evaluation.

### Team member

A project team of 11 medical staff was established, including two head nurses, six physicians, two research nurses qualified by Joanna Briggs Institute (JBI) evidence-based training, and one staff from the quality management department. The educational background of team member was six doctors, three masters, and two undergraduates. The research nurses were responsible for collecting, collating, and summarizing evidence, and the physicians and head nurses were responsible for the validity discussion of evidence. The head nurses were also responsible for translating the best evidence into parameters, training nurses, implementing quality review and improvement. The quality management department staff was responsible for the supervision and advice for the entire study.

### Literature search

The literature search was based on PIPOST model: (1) Patient: patients underwent HSCT; (2) Intervention: early identification, assessment and treatment of TA-TMA; (3) Professional: medical care providers; (4) Outcome: diagnostic criteria, high-risk factors, and early parameters and prognosis of TA-TMA; (5) Setting: hospitals; (6) Types of evidence: guidelines, evidence synthesis, best practice information books, recommended practices, expert consensus, systematic reviews, diagnostic studies, cohort studies, case series studies, etc. according to the evidence 6S pyramid model [14]. Databases included Up To Date, Cochrane, and BMJ Best Practice, and guideline website including GIN, NICE, SIGN, NGC, RNAO, and Medlive Guideline were searched. OVID, Pubmed, EMBase and China Biomedical Literature Database (CBM) were also searched. The searched strategy included "Hematopoietic stem cell transplantation", "thrombotic microangiopathy", "TA-TMA", "transplant-associated thrombotic microangiopathy", "progressive", "early", "at-risk", "high risk". The search timeframe of each database was from January 1980 to January 2020.

### Literature and evidence quality

The quality of literature and evidence was independently evaluated by two investigators and any disagreements were solved by the expert in Evidence-based Medicine.

The quality of guideline was evaluated by British Appraisal of Guidelines Research and Evaluation ii (AGREE ii) criteria [15]. The quality of literatures involved in evidence synthesis, best practice information books, and recommended practice were evaluated according to the study design. The quality of expert consensus, systematic reviews, diagnostic studies, cohort studies, and case series was evaluated in accordance with the quality assessment tools of JBI Evidence-based Health Care Australia.

The quality of evidence was evaluated according to the pre-ranking system of the Australian JBI Evidence-based Health Care Center (2014). The quality of evidence would be upgraded or degraded further according to GRADE principle that graded evidence into Level 1–5 [16]. In case of disagreement from different literature, the determination was made according to the quality of evidence. The coefficient, authority coefficient of six physicians and two head nurses in the project team was 0.863. The evidence was evaluated according to the FAME scale (Feasibility, Appropriateness, Meaningfulness and Effectiveness).

### Healthcare quality review and improvement

The main components of the healthcare quality review and improvement included baseline investigation, improvement action and effectiveness evaluation.

Nurses from Bone Marrow Transplantation Center of the First Affiliated Hospital of Medicine college, Zhejiang University were included in the baseline investigation of the awareness and knowledge of early parameter of TA-TMA. To ensure the reliability of the survey, the electronic questionnaires were anonymously collected and a time limit of 2 min was requested. The gaps of TA-TMA early identification were analyzed based on baseline investigation. The improvement action was subsequently developed and implemented at the Bone Marrow Transplantation Center, First Affiliated Hospital of Zhejiang University School of Medicine.

The effectiveness evaluation of the improvement action consisted of two aspects. One was to investigate the awareness and knowledge of early parameter of TA-TMA among nurses after the implementation of improvement action. Another was to explore the impact of the improvement action on the prognosis of patients underwent HSCT. The occurrence of TA-TMA among patients who underwent HSCT at Bone Marrow Transplantation Center, First Affiliated Hospital of Zhejiang University School of Medicine, were collected before and after improvement action (between 2017–2019 and between 2020–2021, respectively). In addition, the reduce of falls events, one of the common clinical events related to TA-TMA and critical clinical events after HSCT, were evaluated, which was also the one of the critical goals of the nursing departments.

This study was approved by the Ethics Committee of the First Affiliated Hospital of Zhejiang University School of Medicine. Informed consent to participate was waived by the ethics committee for the retrospectively included patients due to the retrospective design; informed consent to participate was obtained from nurses investigated and patients included after improvement action.

### Statistical analysis

Mean ± standard deviation or median (range) was used for continuous variables and number (percentage) for categorical variables. Comparisons were performed using chi-square test or Fisher's exact probability method. *P* < 0.05 was considered statistically different.

## Results

### Analyzed literature

A total of 25 literatures were initially analyzed in this study, containing 1 guideline [17], 1 evidence synthesis [18], 4 expert consensuses [1, 19–21], 10 literature reviews [9, 22–30], 2 diagnostic studies [31, 32], and 7 case series [8, 33–38].

### Literature and evidence quality

The kappa values ​​of the two reviewers in this study were greater than 0.4, indicating a good consistency of the reviewers' opinions. One guideline was included [17], with a scores of six quality evaluation dimensions of 88.89%, 77.78%, 78.13%, 97.22%, 72.92%, and 95.83%, respectively, which was strongly recommended [39]. Additional two literature reviews [40, 41] and 6 case series studies [42–47] were analyzed after tracking literatures in the guideline. The analyzed expert consensuses were answered with "Yes" in quality evaluation, except the consensus by Dvorak et al. [21], where the answer of item 4 was "unclear". The quality evaluation of 12 included literature reviews [9, 22–30, 40, 41] showed answers of " Not applicable" regarding item 2, item 3, item 5, item 6, and item 7, whereas other items were answered "Yes". Considering the exclusion of literature reviews might eliminate certain evidence, the literature reviews were finally included in the best evidence.

One included diagnostic study by Dandoy et al. [31] was answered with "Yes", except for item 3 and item 8, and the other by Schuh et al. [32] was answered with "Yes", except for item 2, item 7, and item 8. Among the 13 analyzed case series, 9 were answered with "Yes" for all items and were included in the best evidence [8, 34–38, 42, 45, 46].

### Evidence-based model

After comprehensively reviewing the evidence and eliminated the parameter not applicable during nursing, early warning model consisting of ten parameters from best evidence was developed (Table 1).

**Table 1:** Evidence-based model evidence-based model for early identification of TA-TMA

Primary	Secondary	Recommendation level
Warning period	1. The median onset time of TA-TMA in children was about 30–45 days after HSCT [30]	Grade-A Recommendation
	2. The onset time of TA-TMA in adults ranged from 13 to 319 days [21, 31], and in the transplant warehouse, the peak time might be 20–25 days after transplantation [36]	Grade-A Recommendation
High-risk characteristics	4. The strongest predictors of TA-TMA were grade II-IV aGVHD and using tacrolimus and sirolimus in combination [38], where the incidence of TA-TMA in aGVHD patients is four times that in patients without aGVHD [25]	Grade-A Recommendation
	5. Patients receiving a ≥ 16 mg/kg dosage of busulfan in the myeloablative regimen [8]	Grade-B Recommendation
Early manifestation	6. Hypertension was usually the first clinical symptom of TA-TMA. Some patients underwent HSCT might develop unexplainable hypertension 10 to 14 days before TA-TMA diagnosis [23, 30]	Grade-A Recommendation
	7. LDH exceeded the upper limit of the normal range and TA-TMA index (LDH/platelet: 1000) ≥ 20 [30]	Grade-A Recommendation
	8. Proteinuria was an essential marker of TA-TMA, and serum creatine was not sensitive in the early stage [36]. The increase of creatine might occur at the late-stage of TA-TMA, indicating the beginning of irreversible kidney damage [23]	Grade-A Recommendation
	9.Unexplained platelet decline ≥ 50% or unexplained hemoglobin decline and reticulocyte increase [1]	Grade-B Recommendation
	10. More than 4% fragmented red blood cells with negative result of the direct antiglobulin test [22]	Grade-A Recommendation

*TA-TMA* transplant-associated thrombotic microangiopathy, *HSCT* Hematopoietic stem cell transplantation, *LDH* Lactate dehydrogenase, *aGVHD* acute graft-versus-host disease

The quality review points were developed according to the evidence-based model for early identification of TA-TMA: (1) To ensure that 100% of nurses could recognize the six parameters of TA-TMA and patients with at least one should be suspected to have TA-TMA: unexplained hypertension, elevated lactate dehydrogenase (LDH), positive urine protein or elevated serum creatine, fragmented red blood cells in blood cells, unexplained decrease in platelets and/or hemoglobin; (2) To ensure the nurses could distinguish high-risk patients: the ≥ 16 mg/kg of busulfan, grade II-IV acute graft-versus-host disease (aGVHD), or the combined use of tacrolimus and sirolimus; (3) To ensure the nurse report timely to the attending doctor after identifying patients with suspected TA-TMA; (4) To ensure the symptoms of patients with suspected TA-TMA improved after intervention; (5) To ensure the education and implementation of bed resting in patients with suspected TA-TMA; (6) To ensure no in-hospital fall occurred in patients with suspected TA-TMA.

### Baseline investigation

The average age of 75 investigated nurses was 28.6 ± 5.9 years. There were 2 deputy chief nurses, 16 chief nurses and 57 regular nurses. There were 2 nurses with master's degree, 64 with bachelor's degree and 9 with associate degree. The response rate and completeness of questionnaire was both 100%.

The awareness rate of all six parameters for TA-TMA was only 26.7%, among which fragmented erythrocyte was all recognized. The awareness rate of unexplained hypertension, LDH, positive urine protein or increased serum creatine, unexplained thrombocytopenia and hemoglobin decline was 46.7%, 34.7%, 41.3% and 44%, respectively. The proportion of nurses who were aware the importance and actually implemented bed resting in patients with suspected TA-TMA was 49.3%.

### Improvement action

Based on the baseline investigation and quality review points, the gaps were identified: (1) TA-TMA has not attracted the attention of transplant medical staff due to the low incidence; (2) The early identification of TA-TMA is complicated and difficult, with the limited time around patients from attending physician and limited experience of the nurses; (3) The awareness and knowledge of parameters for TA-TMA was insufficient among nurses; (4) There is no standard system for observing and reporting TA-TMA; (5) TA-TMA generally occurred during blood cell engraftment when the vigilance of healthcare providers decreased; (6) No caregiver was allowed in the transplantation cabin and companion of all time provided by the nurse was not available. The improvement action was developed according to the identified gaps (Table 2).

**Table 2:** Improvement action

Staff training and assessment	The head nurse is responsible for training the nurses about the best evidence-based early warning model of TA-TMA, including warning period, high-risk characteristics and early manifestation of TA-TMA. The training should be included in the department's training program plan, and conducted during the morning meeting time. The passing rate of the assessment test should reach 100%
Suspected TA-TMA identification	Incorporate the ten items in the model into the nursing routine. Nurses should assess whether the patient was suspected to have TA-TMA based on any of the following: unexplained hypertension, elevated LDH, positive urine protein or elevated serum creatine, fragmented red blood cells, unexplained platelets or hemoglobin decline. The timely report to the attending physician and discussion on the diagnosis of TA-TMA should be done. The corresponding nursing interventions should be carried out for patients diagnosed with or suspected of TA-TMA
Patient education	The department produced a video for patient education, mainly focus on the prevention of fall events in patients with suspected or confirmed TA-TMA. The content included the causes and unpredictability of falls events caused by TA-TMA and possible harm of fall events. Sign informed consent should be obtained from the patients and activities should be carried out in the company of a nurse except for reposing and turning over in bed
Routine healthcare quality review	The prevention of fall events in patients with suspected or confirmed TA-TMA should be a quality improvement project implemented by a well-established quality control team. The team leader should conduct a healthcare quality review after the shift every morning, including whether the identification of TA-TMA, the implementation of the prevention of fall event, and patients' awareness and cooperation. The head nurse should hold a meeting and summary the TA-TMA related events every month, and improve the ability of nurses through retraining

*TA-TMA* transplant-associated thrombotic microangiopathy, *LDH* Lactate dehydrogenase

### Effectiveness of improvement action

The awareness rate of warning period, high-risk characteristics and early manifestation of TA-TMA after improvement action all reached 100%, which was significantly increased compared with 26.7% before improvement action (*P* < 0.001).

Among 500 patients underwent HSCT before improvement action, 14 patients (2.8%) were diagnosed with TA-TMA, among which six (1.2%, 42.9% among patients with TA-TMA) patients had fall events. The fall events in patients with TA-TMA accounted for 66.7% of the fall events during 2017–2019. Five hundred patients underwent HSCT during 2020–2021 were randomly selected. Fifty-six (11.2%) were identified as patients with suspected TA-TMA, among which 49 (9.8%) showed improvement and 7 (1.4%) were diagnosed with TA-TMA. The incidence of TA-TMA was similar before and after improvement action (P = 0.643), while no fall event occurred after improvement action (0 vs. 1.2%, *P* < 0.001).

## Discussion

In this study, we searched and review the best evidence for early identification of TA-TMA in patients underwent HSCT. The nurse-leading early warning model, consisting of warning period, high-risk characteristics and early manifestation of TA-TMA was developed. The gaps were identified from the baseline investigation and the early warning model, and the subsequent improvement action, including staff training and assessment, suspected TA-TMA identification and patient education, was implemented. The awareness and knowledge rate of TA-TMA warning after improvement action among nurses significantly improve. Though the incidence of TA-TMA was similar among patients underwent HSCT before and after improvement action, no fall event occurred after improvement action. The warning model might help provide practical tools for nurses and physicians in the early diagnosis of TA-TMA, especially considering novel therapies are being developing for TA-TMA.

It was reported that 29% of patients with TA-TMA patients had neurological symptoms, 66% had proteinuria, and 32% had new-onset or worsening hypertension [38]. Nevertheless, the common symptoms of TA-TMA often overlapped with other complications after HSCT. The diverse and nonspecific clinical manifestations of TA-TMA contributed to the lack of a gold diagnosis standard. The early identification of TA-TMA remains a challenge in clinical practice, especially for nurses. In this study, the best evidence-based early warning model for TA-TMA was established, using proper methods for literature search and evidence quality assessment. The model consisted of three critical aspects of early identification of TA-TMA. The evidence on warning period could help healthcare providers keep alert at the appropriate time and best utilized the time [48]. The awareness of characteristics among patients with high-risk of TA-TMA and early manifestation of TA-TMA ensured the individual monitor and early identification of TA-TMA for patients underwent HSCT.

Though the pathogenesis of TA-TMA has not been fully understood, the three-hit hypothesis was widely recognized [7]. Once extensive endothelial dysfunction occurs, it might be difficult for patients to benefit from risk factor modification and treatment [49]. The early disruption of the endothelial injury might be the key in the prevention and treatment of TA-TMA, which emphasized on the importance of early identification of TA-TMA. Noticeably, hypertension is usually the first symptom of endothelial dysfunction [21], which could easily and frequently monitored by nurses. In the present early warning model, the variables included in the model could be mostly assessed independently by nurses who frequently checked on the patients’ condition and had chances to timely recognize the development of TA-TMA. In the meantime, the healthcare quality improvement project enabled a deeper communication and collaboration between physicians and nurses. For instance, the decrease in platelet count, which was considered to be a symptom of TA-TMA [1], was common in patients receiving conditioning regimens before transplantation. The discussion and determination of cause of thrombocytopenia could enhance the knowledge acquisition and experience accumulation among nurses.

The incidence of TA-TMA was 3% in 23,665 allogeneic hematopoietic stem cell transplantation reported by the Center for International Blood and Marrow Transplant Research (CIBMTR) [10], which was similar to 2.8% before the healthcare quality improvement project at our center. By the implementation of the improvement action, the awareness and knowledge rate of TA-TMA warning significantly improve and reached 100%. As a result, 56 out of 500 patients underwent HSCT were identified as suspected TA-TMA, and only 7 were finally progressed to the confirmed diagnosis of TA-TMA under appropriate prevention, leading to an incidence of 1.4%. Nevertheless, the difference of incidence before and after the project was not statistically significant, possibly due to low incidence of TA-TMA. In addition, reducing the incidence of falls events among hospitalized patients is one of the critical goals of the nursing departments [50]. Specifically, the engraftment of blood cell after transplantation when patient regained the physical strength might relax the vigilance for fall events. Among patients with TA-TMA, that often manifested with nervous system symptoms, the incidence might be higher and the prognosis might be worse. In our center, no fall event occurred after improvement action, which was significantly improved compared with that before the action. Taken together, the healthcare quality improvement project might demonstrate favorable effect on TA-TMA prevention and the prognosis of patients underwent HSCT.

There were several limitations need to be addressed. Firstly, the healthcare quality improvement project was development based on the baseline investigation in three centers, the universality of such project should be concerned. Secondly, parallel comparator was not available in this study, and the comparison before and after the project might result in unavoidable bias. Finally, the small number of TA-TMA events and lack of survival data limited the analysis, including the adjustment of confounders that are not unique to TA-TMA or TA-TMA risk factors (e.g. previous history of osteoporosis).

In this study, the evidence-based, nurse-leading early warning model and healthcare quality improvement project could enhance the awareness and knowledge of TA-TMA among healthcare providers and might improve the prognosis of patients diagnosed with TA-TMA. The results of this study might also provide benchmarking for future study designs and healthcare improvement project.

## Data Availability

The datasets used and/or analyzed during the current study are available from the corresponding author on reasonable request.
